# Radionuclide Therapy of HER2-Expressing Xenografts Using [^177^Lu]Lu-ABY-027 Affibody Molecule Alone and in Combination with Trastuzumab

**DOI:** 10.3390/cancers15092409

**Published:** 2023-04-22

**Authors:** Yongsheng Liu, Tianqi Xu, Anzhelika Vorobyeva, Annika Loftenius, Vitalina Bodenko, Anna Orlova, Fredrik Y. Frejd, Vladimir Tolmachev

**Affiliations:** 1 Department of Immunology, Genetics and Pathology, Uppsala University, 751 85 Uppsala, Sweden; yongsheng.liu@igp.uu.se (Y.L.); tianqi.xu@igp.uu.se (T.X.); anzhelika.vorobyeva@igp.uu.se (A.V.); fredrik.frejd@affibody.se (F.Y.F.); 2 Affibody AB, 171 65 Solna, Sweden; annika.loftenius@affibody.se; 3 Research Centrum for Oncotheranostics, Research School of Chemistry and Applied Biomedical Sciences, Tomsk Polytechnic University, 634050 Tomsk, Russia; bodenkovitalina@gmail.com; 4 Department of Medicinal Chemistry, Uppsala University, 751 23 Uppsala, Sweden; anna.orlova@ilk.uu.se

**Keywords:** HER2, radionuclide therapy, Affibody molecule, lutetium-177

## Abstract

**Simple Summary:**

Affibody molecules are artificial proteins that can recognize cancer-related molecular abnormalities in the living body. Clinical studies demonstrated that Affibody molecules can be successfully used for radionuclide diagnostics. Targeted radionuclide therapy selectively delivers cytotoxic radionuclides to malignant tumors, thus sparing normal tissues. For radionuclide therapy, Affibody molecules were re-engineered to decrease accumulation in the kidneys. This study has demonstrated that radionuclide therapy using re-engineered Affibody molecules increases the survival of immunodeficient mice bearing human tumors. The therapy was more efficient than the treatment with a monoclonal antibody, which is currently used in clinical practice. The best results were obtained when both the antibody and radiolabeled Affibody molecules were used simultaneously. This work provides a preclinical rationale for a potentially more efficient treatment in HER2-positive cancers.

**Abstract:**

ABY-027 is a scaffold-protein-based cancer-targeting agent. ABY-027 includes the second-generation Affibody molecule Z_HER2:2891_, which binds to human epidermal growth factor receptor type 2 (HER2). An engineered albumin-binding domain is fused to Z_HER2:2891_ to reduce renal uptake and increase bioavailability. The agent can be site-specifically labeled with a beta-emitting radionuclide ^177^Lu using a DOTA chelator. The goals of this study were to test the hypotheses that a targeted radionuclide therapy using [^177^Lu]Lu-ABY-027 could extend the survival of mice with HER2-expressing human xenografts and that co-treatment with [^177^Lu]Lu-ABY-027 and the HER2-targeting antibody trastuzumab could enhance this effect. Balb/C nu/nu mice bearing HER2-expressing SKOV-3 xenografts were used as in vivo models. A pre-injection of trastuzumab did not reduce the uptake of [^177^Lu]Lu-ABY-027 in tumors. Mice were treated with [^177^Lu]Lu-ABY-027 or trastuzumab as monotherapies and a combination of these therapies. Mice treated with vehicle or unlabeled ABY-027 were used as controls. Targeted monotherapy using [^177^Lu]Lu-ABY-027 improved the survival of mice and was more efficient than trastuzumab monotherapy. A combination of therapies utilizing [^177^Lu]Lu-ABY-027 and trastuzumab improved the treatment outcome in comparison with monotherapies using these agents. In conclusion, [^177^Lu]Lu-ABY-027 alone or in combination with trastuzumab could be a new potential agent for the treatment of HER2-expressing tumors.

## 1. Introduction

Targeted therapy using monoclonal antibodies (mAbs) is a useful treatment option for patients with disseminated cancers. For instance, the monoclonal antibodies trastuzumab and pertuzumab targeting human epidermal growth factor receptor 2 (HER2) are currently used in combination with chemotherapy as a standard therapy against HER2-positive breast and gastric cancer [1,2,3]. However, resistance to trastuzumab during therapy is often developed despite the preserved high level of HER2 expression [4]. The addition of a cytotoxic payload to a mAb might further extend the anti-cancer action [5]. The specific binding of mAbs to cell-surface antigens allows antibody-drug conjugates (ADCs) to be selectively delivered to the tumor cells [6]. For example, a HER2-targeting ADC, trastuzumab emtansine (T-DM1), provided a high response rate in patients pre-treated with trastuzumab [7]. However, resistance to the cytotoxic payload may also be developed by tumor cells through a variety of mechanisms [8]. Radionuclides can be considered an alternative cytotoxic payload because they are not associated with multidrug resistance. Radioimmunotherapy (RIT), which is based on the specific transport of radionuclides to tumor lesions by antibodies, has shown a high remission rate when used against tumors of hematopoietic and lymphoid origin [9]. Still, RIT of solid tumors that are typically more radioresistant was less successful. The major issue, in this case, is the large size of antibodies, which affects their penetration into the tumor tissue. In addition, antibodies remain in circulation for a long time, which causes a substantial absorbed dose to bone marrow and narrows the therapeutic window. Smaller tumor-targeting agents would be an attractive alternative to mAbs for targeted radionuclide therapy.

Affibody molecules are engineered scaffold proteins potentially suitable for targeted radionuclide therapy [10,11]. These small proteins are robust and refold after denaturation. Development of high-affinity Affibody binders to several cancer-related molecular targets has been reported. HER2-targeting Affibody molecules have been evaluated in Phase I/II clinical trials [12,13]. These trials demonstrated that injections of Affibody molecules are safe. Moreover, radiolabeled Affibody molecules have shown excellent targeting of HER2-positive breast cancer metastases [12,13]. However, a high re-absorption of Affibody molecules in the proximal tubuli of the kidney leads to high renal absorbed doses [14], which restricts their utility for radionuclide therapy. Reduction of the renal uptake of Affibody molecules is possible by fusion with an albumin-binding domain (ABD) [15]. ABD is a small (5 kDa) robust protein. Proteins, which are fused or conjugated to ABD, bind the host’s albumin in vivo, which prevents or hampers their filtration in glomeruli and reduces their renal uptake [16]. The kidney uptake of the ABD-fused dimeric anti-HER2 Affibody molecule, [^177^Lu]Lu-CHX-A″-DTPA-ABD-(Z_HER2:342_)_2_, was reduced by more than 20-fold compared with the uptake of non-ABD-fused Affibody molecule [^177^Lu]Lu -CHX-A″-DTPA-(Z_HER2:342_)_2_ [15]. Obviously, the prevention of glomerular filtration resulted in a noticeable increase in the activity in blood circulation. Besides, the hepatic uptake of [^177^Lu]Lu-CHX-A″-DTPA-ABD-(Z_HER2:342_)_2_ increased 5.4-fold (48 h after injection) compared to [^177^Lu]Lu-CHX-A″-DTPA-(Z_HER2:342_)_2_. Still, an experimental therapy in mice with HER2-expressing SKOV-3 xenografts demonstrated a dose-dependent survival enhancement of the treated mice. Thereafter, a second-generation ABD-fused HER2-targeting agent Z_HER2:2891_-ABD-C-DOTA (denoted ABY-027) has been developed [17]. This construct contains a monomeric targeting Affibody molecule, Z_HER2:2891_, which is based on a second-generation Affibody scaffold [18]. This targeting moiety has higher stability and is more hydrophilic than its predecessor Z_HER2:342_. A second-generation ABD with femtomolar affinity to human albumin was used for a half-life extension [19]. For site-specific radiolabeling, a unique cysteine was introduced at the C-terminus of ABY-027, and a maleimido derivative of DOTA chelator was conjugated. ABY-027 has an affinity of 74 pM to HER2, subpicomolar affinity to albumin, and has been labeled with lutetium-177 with a high yield. [^177^Lu]Lu-ABY-027 provided a further reduction of renal and hepatic uptake of radioactivity compared to [^177^Lu]Lu-CHX-A”-DTPA-ABD-(Z_HER2:342_)_2_. It was demonstrated that the uptake of [^177^Lu]Lu-ABY-027 in tumors remained high and HER2-specific. An assessment of [^177^Lu]Lu-ABY-027 dosimetry suggested that it is suitable for radionuclide therapy [17]; however, the therapy experiments were not performed so far.

Previous studies have shown that treatment with trastuzumab enhances the radiosensitivity of HER2-expressing cancer cell lines [20]. It was demonstrated that adjuvant trastuzumab reduces the risk of locoregional recurrence in patients with breast cancer who received completed radiotherapy [21]. It would be attractive to combine radionuclide therapy and trastuzumab in the treatment of disseminated HER2-positive cancers. Structural studies demonstrated that trastuzumab and an anti-HER2 Affibody molecule bind to HER2 at different domains [22]. This gives an opportunity for co-treatment by trastuzumab and Affibody-mediated radionuclide therapy.

The goals of this study were to test the hypotheses that a targeted radionuclide therapy using [^177^Lu]Lu-ABY-027 could extend the survival of mice with HER2-expressing human xenografts, and that co-treatment with [^177^Lu]Lu-ABY-027 and trastuzumab could extend the survival of such mice even further. For this purpose, interference of binding of [^177^Lu]Lu-ABY-027 and trastuzumab to HER2-expressing cells in vitro was first evaluated. The effect of co-treatment on xenograft growth and survival of mice was then compared with the effect of treatment using trastuzumab or [^177^Lu]Lu-ABY-027 alone.

## 2. Materials and Methods

### 2.1. General

No-carrier-added ^177^LuCl_3_ was obtained from Curium Pharma (Stockholm, Sweden). An automated gamma-spectrometer (2480 Wizard, PerkinElmer, Waltham, MA, USA) was used for activity measurements. A Storage Phosphor System (CR-35 BIO Plus, Elysia-Raytest, Bietigheim-Bissingen, Germany) was used for activity quantification in instant thin-layer chromatography (iTLC) strips. The Affibody molecule ABY-027 was kindly donated by Affibody AB (Solna, Sweden).

In vitro cell studies were performed using the HER2-expressing ovarian cancer SKOV-3 cells. Cell line was obtained from the American Type Culture Collection (ATCC, Manassas, VA, USA). Cells were cultured in Roswell Park Memorial Institute (RPMI) 1640 medium supplemented with 10% fetal calf serum, 2 mM L-glutamine, 100 IU/mL penicillin, and 100 mg/mL streptomycin.

### 2.2. Labeling Chemistry

For the labeling of ABY-027 with Lu-177, an aliquot of 200 µg ABY-027 was reconstituted in 200 µL of 0.2 M ammonium acetate, pH 6.0, mixed with 22.7 µL of Lu-177 stock solution (500 MBq), and incubated at 65 °C for 30 min. After incubation, small (~1 µL) samples were taken to analyze the radiochemical yield by iTLC. The iTLC strip was eluted with 0.2 M citric acid, pH 2.0.

### 2.3. In Vitro Studies

Binding affinity of [^177^Lu]Lu-ABY-027 to HER2-expressing SKOV-3 cells was evaluated in the absence and presence of 100 nM HSA, using LigandTracer (Ridgeview Instruments AB, Uppsala, Sweden). Briefly, SKOV-3 cells were seeded on a local area of a cell culture dish (Nunclon^TM^, Size 100 mmNUNC A/S, Roskilde, Denmark). [^177^Lu]Lu-ABY-027 with concentrations of 0.25, 0.75, and 2.25 nM was added to the cells to estimate the association rate. The kinetics of association and dissociation were measured in real time, as described in [23]. To evaluate the effect of trastuzumab on binding of [^177^Lu]Lu-ABY-027 to HER2-expressing SKOV-3 cells, 20 µg/mL (135 nM) of trastuzumab was added to the media to mimic the concentration of trastuzumab in the extracellular fluid of mice after injection of clinically used loading dose of trastuzumab (4 mg/kg). The experiment was performed as described above, both in the absence and presence of HSA. The data were analyzed by the InteractionMap software (Ridgeview Diagnostics AB, Uppsala, Sweden) to calculate equilibrium dissociation constant (KD).

### 2.4. In Vivo Studies

The animal experiments were performed in accordance with national legislation on laboratory animal protection (2010/63/EU), and this study was approved by the local Ethics Committee for Animal Research in Uppsala (permit 5.8.18-11931/2020, 28 August 2020).

To evaluate the blocking of ABY-027 binding to HER2-expressing tumors by trastuzumab in vivo, the biodistribution of [^177^Lu]Lu-ABY-027 with or without blocking by trastuzumab was performed in female Balb/c nu/nu mice bearing SKOV-3 xenografts (two groups, four mice per group). The average animal weight was 21.2 ± 1.6 g at the time of the experiment. The average SKOV-3 tumor weight was 0.06 ± 0.04 g. One group of mice was injected subcutaneously with trastuzumab (clinically relevant dosing, 4 mg/kg) one day before the experiment. Both groups of mice were then injected intravenously with 10 µg (270 kBq) [^177^Lu]Lu-ABY-027 per mouse in 100 µL 1% BSA in PBS. At 48 h after injection, mice were euthanized by an intraperitoneal injection of anesthesia (Ketalar: 10 mg/mL, Rompun: 1 mg/mL). Blood, heart, lung, liver, spleen, pancreas, kidneys, tumor, muscle, bone, gastrointestinal tract, and carcass were collected and weighed. Activity of the samples was measured, and the uptake values were calculated as the percentage of injected dose per gram of the sample (%ID/g).

In vivo imaging was performed to confirm the biodistribution data. A total of 2 mice with SKOV-3 xenografts were injected with 23 MBq (10 µg) of [^177^Lu]Lu-ABY-027. The imaging was performed 48 h p.i. using nanoScan SPECT/CT (Mediso Medical Imaging Systems, Hungary), as described in [24]. The data were reconstructed using Tera-Tomo™ 3D SPECT Software.

### 2.5. Experimental Therapy Study

SKOV-3 cells (10^7^ cells per animal) were subcutaneously implanted on the abdomen of female Balb/c nu/nu mice. The treatment of animals was performed seven days after implantation.

**Figure 1:**
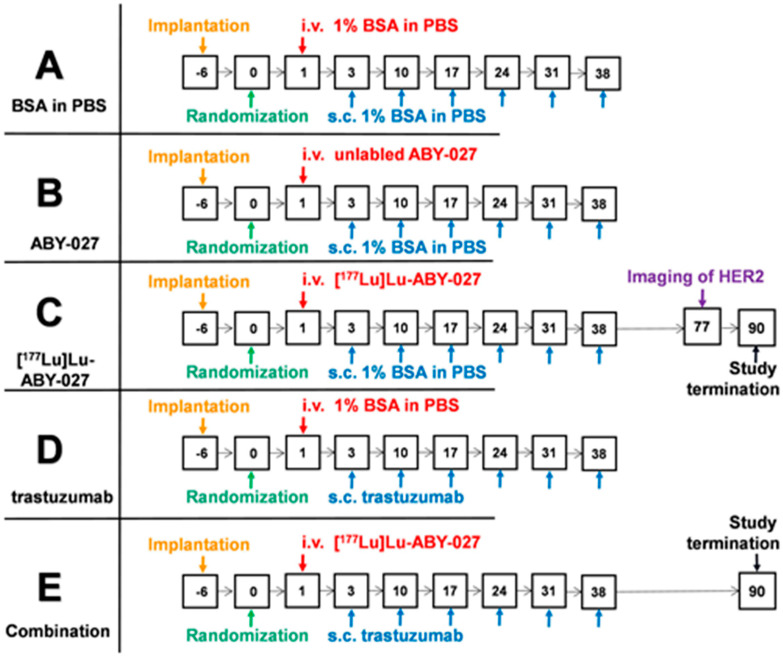
Schematic illustration of the timing of the interventions in treatment experiment.

A schematic illustration of the timing of the interventions in treatment experiment is presented in Figure 1. In one group, mice were injected i.v. with 20 µg (20 MBq) of [^177^Lu]Lu-ABY-027 in 100 µL of 1% BSA in PBS for the 1st week, and then s.c. weekly with 100 µL of 1% BSA in PBS for 5 consecutive weeks. In another group, mice were weekly s.c. injected with trastuzumab with a loading dose of 4 mg/kg for the 1st week and then with 2 mg/kg for 5 consecutive weeks. To evaluate the efficacy of combination treatment with [^177^Lu]Lu-ABY-027 and trastuzumab, 1 group of mice was injected i.v. with 20 µg (20 MBq) of [^177^Lu]Lu-ABY-027 in 100 µL of 1% BSA in PBS and s.c. a loading dose of 4 mg/kg for the 1st week, and then weekly with 2 mg/kg of trastuzumab during 5 consecutive weeks (s.c.). To estimate an effect of non-labeled Affibody molecules tumors growth, a group of mice was injected i.v. with 20 µg of ABY-027 in 100 µL of 1% BSA in PBS for the 1st week and then weekly with 100 µL of 1% BSA in PBS for 5 consecutive weeks. In the vehicle-treated group, mice were injected with 100 µL of 1% BSA in PBS, i.v. for the 1st week, and then s.c. weekly for 5 consecutive weeks. Tumor volumes were measured twice a week, using a caliper to measure the largest longitudinal (length) and transverse (width) diameters. The tumor volume (V) was then calculated using the formula V = 1/2 × (length × width^2^). The tumor volumes at the start of the treatment were 141 ± 25, 139 ± 31, 140 ± 30, 143 ± 23, and 138 ± 27 mm^3^ for mice in groups treated with vehicle, unlabeled ABY-027, [^177^Lu]Lu-ABY-027, trastuzumab and combination of [^177^Lu]Lu-ABY-027 and trastuzumab, respectively. The difference between sizes of tumors was not significant (*p* > 0.05, 1-way ANOVA test at the treatment start). Mice were euthanized if their tumor volume exceeded 1000 mm^3^, if they had bleeding ulcerations, or if they lost more than 15% of their overall weight or more than 10% of their weight within 1 week. This study was terminated 90 days after the first injection, as per the ethical permit, and all mice were humanely euthanized. Kidneys and livers were then collected and fixed with formalin, followed by embedding in paraffin. A pathology examination was performed by an experienced animal pathologist at BioVet AB veterinary medicine lab (Sollentuna, Sweden) on the samples, which were stained with hematoxylin and eosin to detect any histopathologic changes.

To visualize the expression of HER2 during experimental therapy, SPECT/CT scans were conducted of mice with SKOV-3 xenografts using nanoScan SPECT/CT (Mediso Medical Imaging Systems, Budapest, Hungary). On day 77 after treatment initiation, 4 mice from group treated with [^177^Lu]Lu-ABY-027 were injected with 5 µg of [^99m^Tc]Tc-ZHER2:41071 Affibody molecule (16 MBq), and their imaging was carried out at 4 h post-injection.

### 2.6. Statistical Analysis

Data on in vitro studies and biodistribution were analyzed by unpaired 2-tailed t-test or 1-way ANOVA using GraphPad Prism (version 9.3.1 for Windows; GraphPad Software, Inc., La Jolla, CA, USA) to determine significant differences (*p* < 0.05). To elucidate the impact of radionuclide and combination of treatment modalities, the outcomes of therapy in the treatment groups were analyzed using contingency χ^2^-test. For this purpose, the individual outcomes were categorized as a failure (animal had to be euthanized before the last animal in the vehicle-treated control group), limited response (an animal survived longer than the last animal in the vehicle-treated control group but had to be euthanized before the study termination), or response (an animal survived until study termination).

## 3. Results

### 3.1. Radiolabeling and In Vitro Studies

The radiochemical yield of ABY-027 labeling with ^177^Lu was 97.3 ± 2.6% (*n* = 8). In the case of biodistribution and radionuclide therapy experiments, the radiochemical purity was over 98%, and no additional purification was performed. The label was stable under a 1 h challenge with a 500-fold molar excess of EDTA (no measurable release).

**Figure 2:**
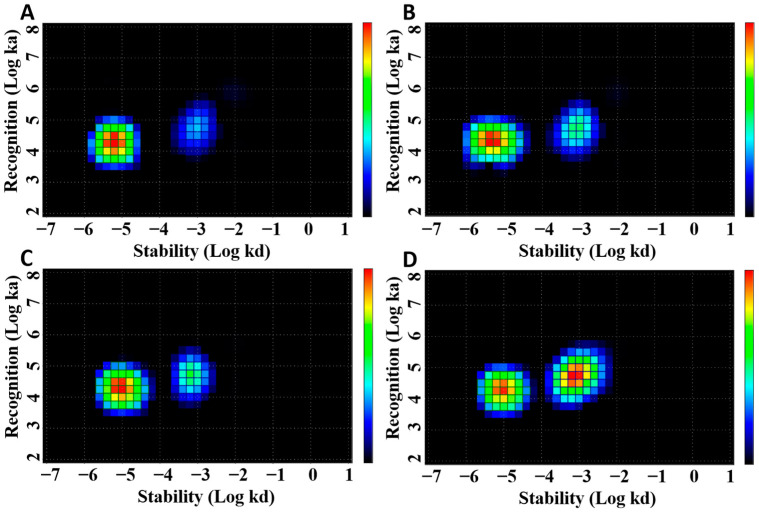
Interaction map of [^177^Lu]Lu-ABY-027 alone (**A**), [^177^Lu]Lu-ABY-027 in the presence of HSA (**B**), [^177^Lu]Lu-ABY-027 in the presence of trastuzumab (**C**), and [^177^Lu]Lu-ABY-027 in the presence of HSA and trastuzumab (**D**) binding to HER2-expressing SKOV-3 cells. Binding was measured with concentrations of [^177^Lu]Lu-ABY-027 at 0.25, 0.75, and 2.25 nM and concentration of trastuzumab at 135 nM.

The data concerning LigandTracer measurements of affinity of [^177^Lu]Lu-ABY-027 binding to living SKOV-3 cells are presented in Figure 2 and Table 1. The pattern of [^177^Lu]Lu-ABY-027 binding was typical for Affibody molecules showing two interactions with different strengths. The strongest interactions in all cases had subnanomolar affinities (apparent equilibrium dissociation constants), and weaker ones were characterized by affinities in the low nanomolar range. The numerical data (Table 1) show that adding HSA did not affect the binding affinity to living HER2-expressing cells. Adding trastuzumab did not influence the interaction with lower affinity but resulted in some reduction of the strength of the high-affinity interaction.

### 3.2. In Vivo Studies

**Figure 3:**
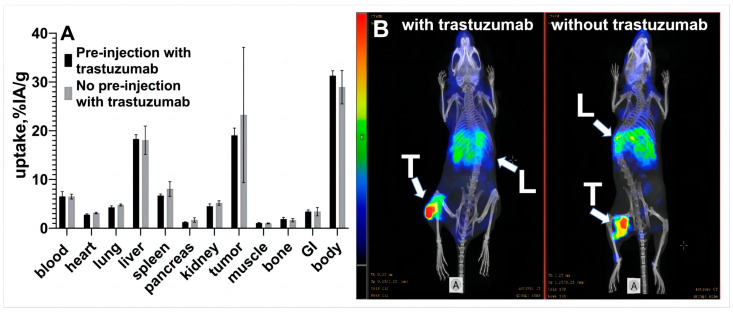
(**A**) Biodistribution of [^177^Lu]Lu-ABY-027 with or without pre-injection of trastuzumab in HER2-expressing SKOV-3 xenografts-bearing Balb/c nu/nu mice at 48 h p.i. Uptake is expressed as %ID/g, which is corrected for decay and presented as average value from 4 mice ± SD. (**B**) Imaging of mice bearing SKOV-3 xenograft 48 h p.i. of [^177^Lu]Lu-ABY-027 with or without pre-injection of trastuzumab using microSPECT/CT. Arrows point at tumors (T) and livers (L). The scale is linear, showing arbitrary units normalized to a maximum count rate.

Results of biodistribution of [^177^Lu]Lu-ABY-027 with or without pre-injection of trastuzumab are shown in Figure 3. There was no significant difference in the uptake of [^177^Lu]Lu-ABY-027 with or without pre-injection of trastuzumab in tumors and among other organs and tissues (Figure 3A). The results of the imaging were in agreement with the biodistribution data (Figure 3B).

**Figure 4:**
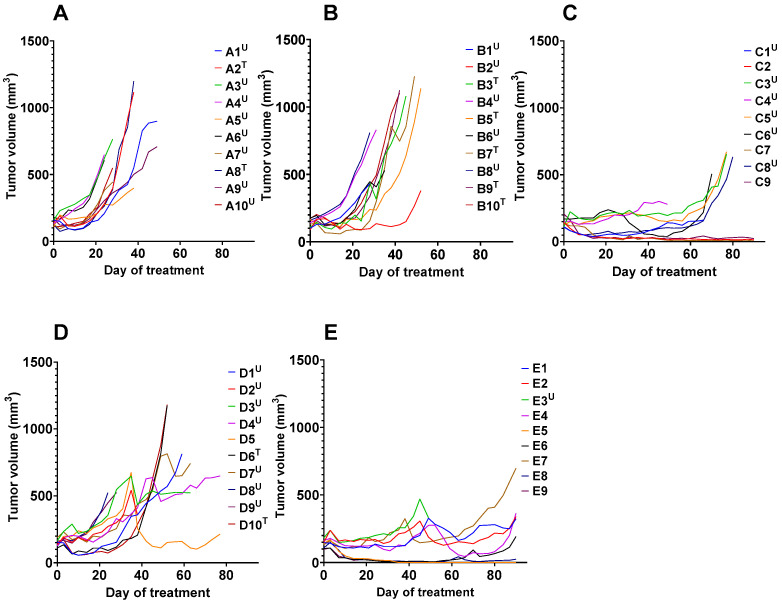
Tumor volume growth curves for individual mice from 5 groups receiving: (**A**) subcutaneous (s.c.) injection of vehicle (1% BSA in PBS) for 6 consecutive weeks, (**B**) intravenous (i.v.) injection of non-labeled ABY-027 (20 µg), (**C**) i.v. injection of [^177^Lu]Lu-ABY-027 (20 µg, 20 MBq), (**D**) s.c. injection of trastuzumab, and (**E**) s.c. injection of trastuzumab together with i.v. injection of [^177^Lu]Lu-ABY-027 (20 µg, 20 MBq). The doses and frequency of trastuzumab injections were 4 mg/kg for the 1st week and 2 mg/kg for the next 5 consecutive weeks. Mice were euthanized when xenograft volume exceeded 1000 mm^3^ or bleeding ulcers were observed. Five groups of mice (ten animals per group) were used in the experiment.

**Figure 5:**
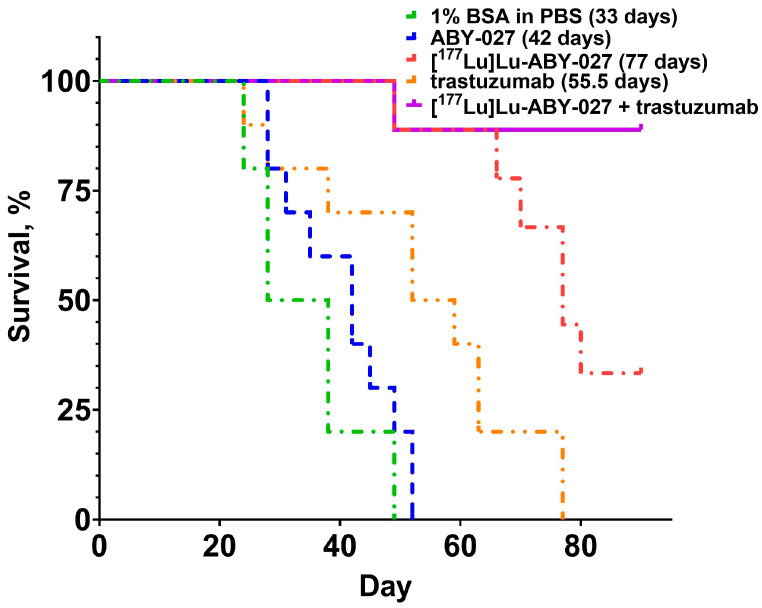
The survival of mice treated with vehicle (1% BSA in PBS), non-labeled ABY-027 (20 µg), [^177^Lu]Lu-ABY-027 (20 µg, 20 MBq), trastuzumab, and combination of trastuzumab and [^177^Lu]Lu-ABY-027 (20 µg, 20 MBq). The doses and frequency of trastuzumab injections were 4 mg/kg for the 1st week and 2 mg/kg for the next 5 consecutive weeks.

**Figure 6:**
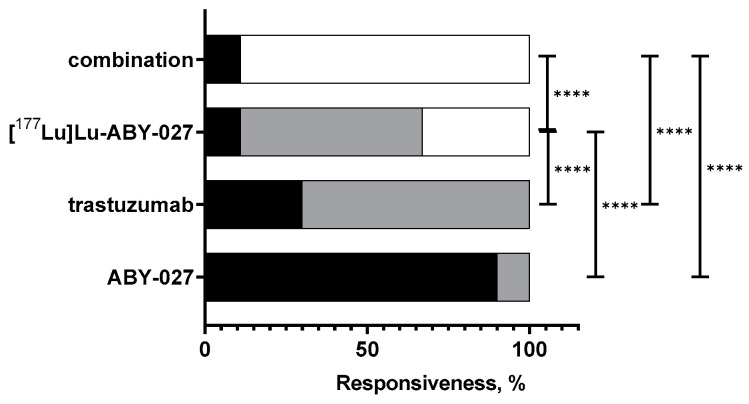
Therapy outcomes. The outcome categories were defined as failure (animal had to be euthanized before the last animal in the vehicle-treated control group, black), limited response (animal survived longer than the last animal in the vehicle-treated control group but had to be euthanized before the study termination, gray), or response (animal survived until study termination, white). The difference between groups was determined using the χ^2^-test. **** corresponds to *p* < 0.0001.

The result of the experimental therapy is presented in Figure 4, Figure 5 and Figure 6. The growth of individual tumors is shown in Figure 4. The tumor volume doubling time was 21 ± 5 days in mice treated with the vehicle and 25 ± 9 days in the mice treated with non-labeled ABY-027. The last animals were sacrificed by days 49 and 52 in the groups treated with vehicle and cold ABY-027, respectively. Tumor growth of mice in the group treated with trastuzumab followed a pattern similar to the pattern for the groups treated with vehicle or cold ABY-027, but with some delay of the tumor growth. The last animal in the group treated with trastuzumab was sacrificed by day 77, and the median survival of mice treated with trastuzumab was 55.5 days, which was significantly longer than the median survival of mice treated with vehicle (*p* < 0.005, Mantel–Cox test) or unlabeled ABY-027 (*p* < 0.05, Mantel–Cox test). The tumor growth patterns in the groups treated with [^177^Lu]Lu-ABY-027 or its combination with trastuzumab were different compared with patterns in the groups treated with vehicle or cold ABY-027. The mean tumor volume was significantly reduced in groups treated with [^177^Lu]Lu-ABY-027 or its combination with trastuzumab from day 14 compared to that in the group treated with vehicle (*p* < 0.05, 1-way ANOVA with Bonferroni correction for multiple comparisons). After some delay, some tumors resumed growth. The median survival of mice treated with [^177^Lu]Lu-ABY-027 alone was 77 days, which is significantly (*p* < 0.01, Mantel–Cox test) longer than the survival of mice treated with trastuzumab alone. A total of 3 animals out of 9 in the group treated with [^177^Lu]Lu-ABY-027 and 8 of 9 in the group treated with [^177^Lu]Lu-ABY-027/trastuzumab combination were alive at the study termination (day 90). Tumors in two animals treated with the combination therapy had disappeared completely by the time of study termination. The mice treated with a combination of [^177^Lu]Lu-ABY-027 and trastuzumab did not reach median survival at the study termination (day 90), thus the survival was significantly longer compared to mice treated with trastuzumab (*p* < 0.05, Mantel–Cox test) or [^177^Lu]Lu-ABY-027 (*p* < 0.0005, Mantel–Cox test) alone (Figure 5).

The effect of radionuclides in the therapy was clearly elucidated by contingency analysis of therapy outcomes (Figure 6). The outcomes of treatment with [^177^Lu]Lu-ABY-027 or its combination with trastuzumab were significantly (*p* < 0.0001, χ^2^-test) different from the outcome of therapy using unlabeled ABY-027. In addition, the outcome of the treatment with [^177^Lu]Lu-ABY-027 was significantly (*p* < 0.0001, χ^2^-test) different and more favorable than the outcome of the treatment with trastuzumab alone. The outcome of the combination of [^177^Lu]Lu-ABY-027 and trastuzumab differed significantly (*p* < 0.0001, χ^2^-test) from the outcomes of monotherapies with these agents and provided enhanced survival.

**Figure 7:**
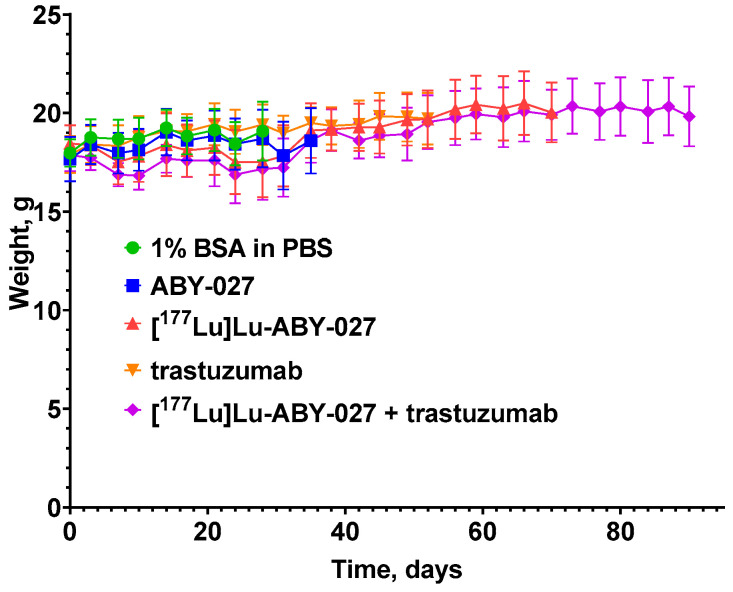
The average body weight of mice treated with vehicle (1% BSA in PBS), non-labeled ABY-027 (20 µg), [^177^Lu]Lu-ABY-027 (20 µg, 20 MBq), trastuzumab, and combination of trastuzumab and [^177^Lu]Lu-ABY-027 (20 µg, 20 MBq). The doses and frequency of trastuzumab injections were 4 mg/kg for the 1st week and 2 mg/kg for the next 5 consecutive weeks.

The mice tolerated the experimental therapies well. The average mice weight did not differ significantly between the treated and control groups (Figure 7). However, one mouse in the group treated with [^177^Lu]Lu-ABY-027 and one mouse treated with a combination of trastuzumab and [^177^Lu]Lu-ABY-027 experienced weight loss beyond humane endpoints and had to be euthanized during experimental therapy. Subsequent pathology analysis showed that these animals suffered from a bacterial infection. Therefore, these animals were excluded from further analysis. According to pathology investigation, there were no toxic effects on the kidney or liver from the different treatments.

**Figure 8:**
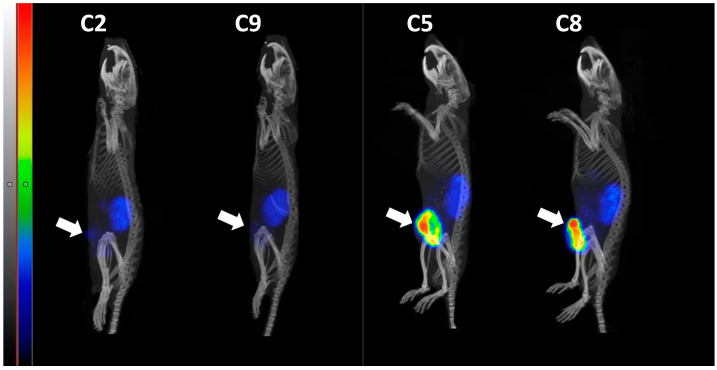
The SPECT/CT imaging using [^99m^Tc]Tc-ZHER2:41071 (maximum intensity projection, sagittal view) of mice after treatment with [^177^Lu]Lu-ABY-027. Imaging was performed 4 h after injection of [^99m^Tc]Tc-ZHER2:41071 at day 77 after treatment started. The arrows point at the tumor implantation sites.

Radionuclide molecular imaging of HER2 expression using [^99m^Tc]Tc-ZHER2:41071 was performed in two mice with tumors with a minimal volume after [^177^Lu]Lu-ABY-027 treatment (C2 and C9) and in two mice with tumors (C5 and C8), which resumed their growth after [^177^Lu]Lu-ABY-027 treatment (Figure 8). The growing xenografts were clearly visualized, and the volume with the high accumulation was within the tumor volume delineated by CT imaging. The tumor uptake exceeded the uptake in any other organs in these animals. On the other hand, there was a very faint uptake in the xenograft implantation sites when the tracer was injected in mice responding to therapy with [^177^Lu]Lu-ABY-027.

## 4. Discussion

Harnessing the excellent tumor-targeting properties of Affibody molecules for radionuclide therapy requires solving the problem of their high uptake and retention in kidneys. For this purpose, we evaluate several strategies. In Affibody-mediated pretargeting, a therapeutic radionuclide is coupled to a small hapten with low reabsorption in kidneys [25,26]. The use of non-residualizing ^131^I [27] and ^188^Re [28] labels does not prevent reabsorption but promotes the very rapid release of radiometabolites of Affibody molecules from kidneys. A fusion with ABD and subsequent binding to albumin in vivo makes an Affibody-containing therapeutic construct bigger than 65 kDa and prevents or reduces its passage through the glomerular membrane [15]. This approach enabled radionuclide therapy using the first generation of anti-HER2 Affibody molecules with a random coupling of chelators [15]. The fusion with ABD was also a fruitful way to reduce renal uptake and enabled preclinical radionuclide therapy using another scaffold protein, ADAPT6 [29]. ABD-fusion was also instrumental in the delivery of anti-cancer drugs using HER2-specific Affibody molecules [30] or blocking proliferative signaling by HER3-specific Affibody molecules [31]. In these cases, fusion with ABD increased the bioavailability of the targeting constructs. Development of the second-generation anti-HER2 Affibody molecule ABY-027 for radionuclide therapy added advantages of a more stable tumor-seeking domain, higher affinity to albumin, and site-specific conjugation of the radionuclide [17]. An experience with Affibody molecules for imaging suggested that the positioning of a chelator might affect the biodistribution appreciably and finding the optimal position improves their targeting properties [32]. Therefore, variants of ABY-027 with different positions of the [^177^Lu]Lu-DOTA complex have been evaluated lately. However, placement of DOTA at the N-terminus of such a construct resulted in the renal uptake of 23.2 ± 1.8 %ID/g at 48 h after injection [33], which is much higher than the uptake of [^177^Lu]Lu-ABY-027 in this study (4.51 ± 0.48 %ID/g). In another study, we have positioned the chelator on helix 1 of ABD to ensure minimal interference with binding to albumin, which is mediated by amino acids on helices 2 and 3 [24]. However, the renal uptake of this variant, [^177^Lu]Lu-ABY-271 (12.5 ± 1.0 %ID/g at 48 h), was 2-fold higher than the uptake of [^177^Lu]Lu-ABY-027 during direct in vivo comparison. Thus, the molecular design of ABY-027 seems to be the best for the moment.

A possible way to improve treatment outcomes further is to combine two targeted therapies at the same time. This strategy is utilized in clinical practice by combining trastuzumab and pertuzumab (binding to different domains of HER2) to treat advanced HER2-expressing breast cancer [34]. The use of therapeutics with different modes of action might additionally increase treatment efficacy and prevent resistance development [35]. An important aspect would be the combination of targeting agents with different toxicity profiles. In such a case, the anti-cancer effect would be additive or, hopefully, supra-additive, but normal tissue toxicity of different targeting agents would not be additive. Co-targeting using [^177^Lu]Lu-ABY-027 and trastuzumab should meet this requirement. The strongest side effect of trastuzumab is cardiotoxicity [36], but dosimetry predicts bone marrow toxicity as the dose-limiting for [^177^Lu]Lu-ABY-027 [17]. A noticeable accumulation of [^177^Lu]Lu-ABY-027 was also detected in the liver, but dosimetry calculations [17] suggest that the absorbed dose to the liver should be approximated five-fold lower than the absorbed dose to the tumor. With the accepted absorbed dose to the liver up to 40 Gy [37], we could expect an anti-tumor effect of [^177^Lu]Lu-ABY-027 without hepatic toxicity. The critical requirement would be that these two targeting agents do not interfere with each other’s binding to the molecular target, HER2.

It has been demonstrated earlier that the HER2 targeted Affibody molecule forming the basis of this study and trastuzumab bind to different epitopes [22] at the adjacent subdomains of the extracellular domains of HER2. In vitro and in vivo studies have shown that trastuzumab does not block the binding of the monomeric form of anti-HER2 Affibody molecules belonging to the first generation [38]. Later, an Affibody-based pretargeting system, which utilizes an anti-HER2 Affibody molecule-peptide nucleic acid (PNA) chimera as a pretargeting agent, was evaluated [26]. Such a chimera is bulkier than the monomeric form of an Affibody molecule. In this case, some small but significant reduction of its binding to HER2-expressing cells in vitro was observed when a 200-fold molar excess of trastuzumab was added. However, trastuzumab did not influence the pre-targeted delivery of Lu-177 to tumors in vivo, and the combined treatment with radionuclide pretargeting and trastuzumab was more efficient than any of these therapies alone. ABY-027 bound to HSA has a substantially larger molecular weight than the Affibody–PNA chimera. Therefore, we might expect an interference also in the case of [^177^Lu]Lu-ABY-027, but it was difficult to predict the magnitude of such interference. Hence, we evaluated the impact of trastuzumab on the binding of [^177^Lu]Lu-ABY-027 to HER2-expressing cells (Figure 2 and Table 1). We have found only a mild decrease in the affinity of the strongest interaction of [^177^Lu]Lu-ABY-027 with HER2 expressed by living SKOV-3 cells. Still, even reduced affinity remained in the picomolar range, which is well compatible with in vivo targeting. Remarkably, the addition of albumin had no impact on [^177^Lu]Lu-ABY-027 binding strength, although an association with such bulky protein might create a steric hindrance in binding to the molecular target. No blocking effect of trastuzumab was observed in the in vivo biodistribution of [^177^Lu]Lu-ABY-027 in SKOV-3 xenografts at 48 h p.i. with or without pre-injection of trastuzumab (Figure 3). This indicates that the clinical dose of trastuzumab of 2 mg/kg should not affect the binding of [^177^Lu]Lu-ABY-027 to HER2 in SKOV-3 xenografts.

The experimental therapy demonstrated that the treatment of HER2-expressing human xenografts in mice using [^177^Lu]Lu-ABY-027 extends the survival of animals (Figure 5 and Figure 6). While the median survival of mice treated with the vehicle was 33 days, the median survival of mice treated with [^177^Lu]Lu-ABY-027 was 77 days. The effect was clearly linked with the targeted delivery of Lu-177 to the tumors since the median survival of mice treated with unlabeled ABY-027 was significantly shorter (42 days), and there was a significant difference between outcomes of therapies using unlabeled and labeled ABY-027 (Figure 6). Notably, the use of [^177^Lu]Lu-ABY-027 in this model was more efficient than trastuzumab administered in the same way as the clinical protocol (Figure 5 and Figure 6), as it provided longer median survival and more favorable therapy outcome. An important finding is that the combination of [^177^Lu]Lu-ABY-027 and trastuzumab provides better therapy outcomes (Figure 6) than monotherapies using these agents. Indeed, the majority of animals in the combination therapy group were alive at the study termination, and tumors disappeared in two mice. Thus, combination therapy is a way to increase the anti-cancer effect of [^177^Lu]Lu-ABY-027 without increasing normal organ toxicity. Ideally, an additional control with a negative HER2 expression should be included to demonstrate that the anti-tumor effect is HER2 mediated. However, this would require HER2-negative xenografts having the same growth rate as SKOV-3 to detect a significant difference in median survival. Finding such xenografts would require multiple testing in mice, which contradicts current ethical guidelines aiming at the reduction of the number of animals in biological experiments. Therefore, this experiment was omitted. Another possible control would be a homologous construct containing Affibody molecule, which does not bind to HER2. A therapy using a construct of this type has been tested as a control for the therapy of SKOV-3 xenografts using ABD-fused Affibody molecule of the first generation, [^177^Lu]Lu-CHX-A”-DTPA-ABD-(Z_HER2:342_)_2_ [15]. The results of that study have clearly demonstrated that specific targeting of HER2 is a prerequisite for an efficient treatment of such xenografts. Thus, this control was also not used in this current study to reduce the number of tumor-bearing mice.

Monitoring the therapy course is essential for making the treatment outcome. Particularly, a preserved level of HER2 expression might be essential when the combination therapy targets HER2. Our radionuclide imaging experiment (Figure 8) shows that [^99m^Tc]Tc-ZHER2:41071 Affibody molecule can be used for monitoring HER2 expression during such a therapy. Recently, [^99m^Tc]Tc-ZHER2:41071 was assessed in a Phase I clinical study (ClinicalTrials.gov Identifier: NCT05203497) and demonstrated the capacity to discriminate between breast cancer tumors with high and low HER2 expression levels (Tolmachev, unpublished data).

Taking into account that the combination treatment is optimal with the drugs having different mechanisms of action, possible additional candidates for combined therapy with [^177^Lu]Lu-ABY-027 would be trastuzumab deruxtecan and trastuzumab-emtansine. Trastuzumab deruxtecan exerts its anti-cancer effect by inhibiting topoisomerases and emtansine by blocking the formation of tubulin, which causes mitotic arrest and cell death, i.e., their action is different from the action of ionizing radiation. At the same time, the hematologic toxicities of these drugs are relatively mild [39,40].

A promising way to increase the efficacy of targeted radionuclide therapy for cancer would be to use alpha-emitting radionuclides. Advantages of such nuclides include the short range of alpha-particles, which limits toxicity to non-targeted tissues, and very high linear energy transfer, which enhances the probability of DNA double-strand breaks and deactivation of targeted cells [9]. Emerging clinical data indicate that targeted alpha-therapy might be efficient and reasonably safe in the treatment of castration-resistant prostate cancer [41,42]. It would also be attractive to apply such an approach to the treatment of HER2-expressing breast cancers, which developed resistance to a treatment by antibody and antibody-drug conjugates. ABY-027 could be a suitable option because the DOTA chelator enables stable labeling with such promising alpha emitters as ^225^Ac and ^227^Th [43].

## 5. Conclusions

Radionuclide therapy using [^177^Lu]Lu-ABY-027 increases the survival of mice with HER2-expressing xenografts. The therapy is more efficient than treatment with trastuzumab alone. Co-treatment with [^177^Lu]Lu-ABY-027 and trastuzumab improved the therapy outcome even further and provided some tumor remissions. No toxicity in the kidney or liver was found in the different treatment groups compared to the vehicle control group. This makes the combination of trastuzumab and [^177^Lu]Lu-ABY-027 radionuclide therapy a promising candidate for clinical translation.

## Figures and Tables

**Table 1:** Equilibrium dissociation constants (K_D_) for interaction between [^177^Lu]Lu-ABY-027 and HER2-expressing SKOV-3 cells in presence and absence of HSA and/or trastuzumab analyzed using InteractionMap of LigandTracer Sensorgrams.

Parameter	K_D1_ (pM)	K_D2_ (nM)
[^177^Lu]Lu-ABY-027 only	382.5 ± 53.0	15.7 ± 2.5
[^177^Lu]Lu-ABY-027 with HSA	324.5 ± 75.7	16.5 ± 2.0
[^177^Lu]Lu-ABY-027 with trastuzumab	502.5 ± 46.0	14.5 ± 0.1
[^177^Lu]Lu-ABY-027 with trastuzumab and HSA	533.5 ± 43.1	14.2 ± 2.3

## Data Availability

The data generated during this current study are available from the corresponding author upon reasonable request.
